# Bibliography

**Published:** 1896-06

**Authors:** 


### Bibliography.

LEHRBUCH DER CONSERVIRENDEN ZAHNHEILKUNDE. Von W. D. Miller, a.o., Professor an der Universität Berlin: Mit 420 Abbildungen. Verlag von Georg Thieme, Leipzig, 1896.

Professor Miller in this volume presents the dental profession in Germany a book on operative practice. It is difficult to harmonize this new character with what has previously been accomplished by this distinguished and tireless worker. It is rare to find one interested in pure science capable of bending to the seemingly petty details of operative work with pleasure or even contentment, and still more would it seem difficult for such a person to write a book on such subjects. It is to Professor Miller's honor that he is thus capable of unbending and giving of his practical knowledge for the benefits of students and others who may require it.

The first chapter, after the introduction, is devoted to the treatment of the hard tooth tissues without filling. This includes several well known processes. He gives due credit to Dr. Stebbins for his work in introducing nitrate of silver, published in the INTERNATIONAL DENTAL JOURNAL, 1891. This is followed by methods of separation after the mode adopted by Dr. Arthur.

The second chapter is devoted to "The Filling of the Teeth." Filling-material occupies necessarily many pages. This is followed by sterilization, handling of the instruments, and from these subjects, by proper gradations, with full and explicit directions, is the student led up to the preparation of cavities and filling by various methods in crown and root-canals.

The treatment of a number of pathological conditions closes the book of 416 pages. For the purpose intended, that of a text-book to students in operative work in Germany, it is certainly practical and shows the author to be one of those many-sided but rare men willing to give aid in any direction when most needed.

ELECTRICITY IN ELECTRO-THERAPEUTICS. By Edwin J. Houston, Ph.D., and A. E. Kennelly, Sc.D. New York: The W. J. Johnston Company, 253 Broadway, 1896.

This compact book of four hundred and two pages is, as expressed by the authors, "intended to meet a growing demand which exists not only on the part of general medical practitioners, but also on that of the general public, for reliable information respecting such matters in the physics of electricity applied to electro-therapeutics as can be readily understood by those not specially trained in electro-technics."

There are but few men, it is presumed, better trained for such a work than Professors Houston and Kennelly, and, therefore, those who may feel that they require such a book will find theories, instruments, and processes fully and clearly explained.

Electricity, in its various forms, has become so much a part of medical therapeutics that the practitioner in the several branches of the healing art must make himself intelligent upon it, and so familiarize himself with its technicalities that he will be able to use the current with safety and precision.

The present prospect is that in the very near future a dental office not fitted with all the electrical appliances necessary for use in running machinery, lighting the oral cavity, the reduction of sensitive dentine, etc., will be regarded as being very imperfectly furnished. It is, therefore, quite essential that dentists should begin the study of electrical science, and for a beginner no better book could be taken in hand, as it covers all the important knowledge necessary for a dentist to acquire in the direction of his daily practical work.

DENTAL PATHOLOGY AND PRACTICE. By Frank Abbott, M.D., Professor of Dental Histology, Surgery, and Therapeutics in the New York College of Dentistry, etc. With ninety-seven illustrations. Philadelphia: S. S. White Dental Manufacturing Company, 1896.

This work of two hundred and forty pages is a somewhat unexpected addition to the literature of the subjects treated, as the writer was not aware that any such contribution was in anticipation. The unheralded birth of a new book is not, however, a fault but rather a subject for praise, as thereby undue disappointment is avoided.

The author makes no pretension to having covered the entire subject of dental pathology, for he says, "Such an exhaustive, treatise would assume proportions which would discourage any but the most ardent student in dental surgery."

The book opens with an abstract from Bödecker's "Dental Anatomy and Pathology," giving the views of Heitzmann and Bödecker on development of teeth. This seems to the reviewer to be an unsatisfactory method for an author to pursue. The reader anticipates, upon opening a book of this kind, to, at least, have the author's views upon all topics, and that these should occupy the prominent place in the production.

From Chapter II. to IX., one hundred and two pages, the following subjects are considered: Odontoblasts in their Relation to Developing Dentine, Growth of Enamel, Teeth of the Lower Jaw at Birth, Congenital Defects of Enamel, Studies of the Pathology of Enamel, etc., Caries of the Human Teeth, Children's Teeth and their Treatment, Microscopical Studies upon the Absorption of Roots of Temporary Teeth. It will be seen by these quotations from headings that the subjects considered have been those which have largely occupied the attention of the author for some years; indeed, these chapters are the papers read upon various occasions upon the topics named. The illustrations accompanying the text have been made familiar by previous production in the dental periodicals. These, while carefully and artistically prepared, do not satisfy the critical mind; in fact, are out of date as compared with the photo-micrographic work which, while oftentimes imperfect, leaves no room for cavilling. The author has given in the chapter on "Caries of the Human Teeth" three pictures of this character: 1. Carious Enamel (longitudinal section.) 2. Carious Dentine (longitudinal section.) 3. Carious Dentine (cross section). Whether these represent the views of the author or those of Miller must, for the present, be left to the intelligence of the reader, but they certainly do present more satisfactorily the conditions observed under the microscope than any pencil can give held by human hands.

The chapter following those named is upon filling teeth. Why this is introduced into a work on dental pathology will have to be answered by the author, but it seems to the reviewer that the time has passed when everything pertaining to dentistry can be included under one general heading. Dentistry has grown beyond that adolescent period, and each work should be strictly confined to its proper subjects.

The author does not favor the rubber dam, for he writes, "After having tried more or less faithfully every devise ever brought to my notice for keeping moisture out of cavities during operations, I finally returned to the use of small pieces of linen," but for building up crowns with gold he still has use for the rubber dam.

In the chapter on "Alveolar Abscess" there appears the following sentence: "In the great majority of cases the successful treatment of alveolar abscess is one of the simplest operations we have in dental surgery." This is a somewhat surprising statement in view of the fact that an abscess at the apex of any root means a necrotic condition of the cemental tissue, and that, in its turn, means a continued irritation and an incurable condition, except by excision of the dead portion, a process not mentioned.

The author is not disposed to call pyorrhœa a disease, but rather a condition. His idea is that it begins "in the mouths of children who habitually neglect their teeth," and that by forty years, "the teeth will have become loose, their sockets nearly or quite destroyed." Another cause of pyorrhœa is "mercurial poisoning." Excessive deposits of calcareous matter on the teeth are not, in his opinion, a cause of pyorrhœa. The treatment is mainly the "removal of all foreign substances from the roots of teeth" and "nature, 'the great restorer,' will do the healing of the soft parts."

The chapter on "Facial Neuralgia" is a lengthy one. That on

"Hyperostosis of Roots of Teeth" was published in the *Dental Cosmos* of 1886, and is given entire here, with the illustrations.

Four other chapters conclude the book: Conditions of Patients during which Severe Dental Operations should be avoided, Stomatitis, etc., Contribution to the Knowledge of Tumors of the Jaw, Senile Atrophy of the Upper Jaw.

While the critic cannot quarrel with an author for declining to consider, in a book devoted to dental pathology, all the subjects usually regarded as being included in that branch of dental teaching, it does seem as though this book, nevertheless, is not properly named, inasmuch as it is quite as remarkable for the things left unconsidered as for the character of those for which the author desires special attention.

It is to be regretted that the book is not of a more practical character. Dental teaching to-day needs a work of this kind on dental pathology more than upon any other subject; but it is feared this cannot become a text-book in colleges. Its place will be in the library as a work of reference.

